# Implementation of ultra-hypofractionated radiotherapy for breast cancer in the Netherlands in 2020–2023, using registry data and questionnaires

**DOI:** 10.1186/s13014-025-02669-w

**Published:** 2025-06-12

**Authors:** Anouk H. Eijkelboom, Eva J. A. van Beek, Marcel R. Stam, Paulien Westhoff, Marissa C. van Maaren, Margriet G. A. Sattler, Enja J. Bantema-Joppe, Marcel Verheij, Desirée H. J. G. van den Bongard, Sabine Siesling, on behalf of the NABON-COVID-19 Consortium and the COVID and Cancer-NL Consortium, E. J. T. Luiten, A. E. van Leeuwen-Stok, C. Guerrero Paez, M. E. M. M. Bos, L. B. Koppert, M. J. Hooning, L. J. Boersma, M. L. Smidt, S. Linn, M. T. F. D. Vrancken Peeters, M. K. Schmidt, C. P. Schröder, H. M. Verkooijen, D. H. J. G. van den Bongard, M. Bessems, A. H. Honkoop, P. J. Westenend, S. van der Velde, C. W. Menke-van der Houven van Oordt, E. Manten-Horst, N. T. van Ravesteyn, J. C. Korevaar, J. Verloop, E. J. M. Siemerink, T. van Dalen, A. W. G. van der Velden, M. A. M. Mureau

**Affiliations:** 1 Department of Research and Development, Netherlands Comprehensive Cancer Organisation (IKNL), Boven Clarenburg 2, 3511 CV Utrecht, The Netherlands; 2 Department of Health Technology and Services Research, Technical Medical Centre, University of Twente, Drienerlolaan 5, 7522 NB Enschede, The Netherlands; 3 Radiotherapiegroep, Wagnerlaan 47, 6815 AD Arnhem, The Netherlands; 4 Department of Radiation Oncology, Radboud University Medical Center, Geert Grooteplein Zuid 32, 6525 GA Nijmegen, The Netherlands; 5 Department of Radiotherapy, Erasmus MC Cancer Institute, University Medical Center Rotterdam, Dr. Molewaterplein 40, 3015 GD Rotterdam, The Netherlands; 6 Department of Radiation Oncology, Radiotherapeutisch Instituut Friesland, Borniastraat 36, 8934 AD Leeuwarden, The Netherlands; 7 Department of Radiation Oncology, Netherlands Cancer Institute, Antoni Van Leeuwenhoek, Plesmanlaan 121, 1066 CX Amsterdam, The Netherlands; 8 Department of Radiation Oncology, Amsterdam UMC, De Boelelaan 1117, 1081 HV Amsterdam, The Netherlands

**Keywords:** Breast cancer, Radiotherapy, Questionnaires, Registries

## Abstract

**Background:**

This study investigated the implementation of ultra-hypofractionated radiotherapy (i.e. 5 fractions) in DCIS and early-stage breast cancer, factors associated with its use, and variation across radiotherapy institutes.

**Methods:**

Registry and questionnaire data were used. Registry data included data from the Netherlands Cancer Registry and the NABON Breast Cancer Audit-Radiotherapy (NBCA-R). Women eligible for 5 fractions were included. Trends and variation were visualised using trendlines and case-mix adjusted boxplots. Logistic regression was applied to investigate which factors were associated with the use of 5 fractions. In April 2024 a questionnaire was distributed among radiotherapy institutes to identify facilitators and barriers for implementation.

**Results:**

The current study included 16,115 women. In 2020, 18.5% of the eligible women received 5 fractions, compared to 60.8% in 2023. The lowest variation between radiotherapy institutes was found in 2023 (median: 60.4%, interquartile range: 53.3–70.6%). Age, tumour grade, multifocality, (y)pT, (y)pN, radiotherapy target volume, type of radiotherapy institute, and start year of radiation were associated with the chance of receiving 5 fractions. Sixteen out of the 19 radiotherapy institutes completed the questionnaire, showing variation in age and radiotherapy target volume for which the schedule was used. Most institutes mentioned no barriers for using 5 fractions. Questionnaire data confirmed the trendline finding that national consensus meetings were essential for largescale implementation.

**Conclusions:**

The use of ultra-hypofractionated radiotherapy has increased during the past four years, with reduced variation across Dutch institutes. Registry and questionnaire data indicated that national consensus meetings were instrumental in driving implementation.

**Supplementary Information:**

The online version contains supplementary material available at 10.1186/s13014-025-02669-w.

## Background

Postoperative radiotherapy aims to reduce locoregional recurrence and breast cancer mortality rates in women with breast cancer [5, 6]. Until 2010, patients received 25 fractions of 2 Gray (Gy). A boost could be added to further enhance local control rates for high-risk tumours [17]. In later years, several studies showed that increasing the dose per fraction and reducing the number of fractions, called hypofractionation (13–16 fractions of 2.66–3.2 Gy), resulted in a similar or even improved effect on locoregional recurrences rates, overall survival, and cosmetic outcomes [8, 10, 12, 15, 16, 18, 19].

In March 2020, at the start of the COVID-19 pandemic, the use of ultra-hypofractionated radiotherapy (i.e. 5 fractions of 5.2 or 5.7 Gy) was recommended [3]. These recommendations were based on preliminary results of the FAST- and FAST-Forward trial and were developed in collaboration with European opinion leaders and researchers from these trials [1, 2]. The results of these two randomised controlled trials were published in April and July of 2020. The FAST trial showed that, after 10 years of follow-up, 28.5 Gy in 5 once-weekly fractions was non-inferior to 50 Gy in 25 fractions in terms of normal tissue effects in the breast [1]. The FAST-Forward trial showed that, after 5 years of follow-up, 26 Gy in 5 once-daily fractions was non-inferior to 40 Gy in 15 fractions in terms of local tumour control. Additionally, it was shown to be safe in terms of normal tissue effects [2].

A previous study of our group, using data from 2020–2021, showed that the 5-fraction schedule was first used at the start of the COVID-19 pandemic [7]. A broader implementation was seen after the meeting of the National Platform Radiotherapy Breast Cancer (LPRM) in November 2020. The LPRM defines evidence-based recommendations during meetings with representatives of all Dutch radiotherapy institutes. In November 2020 they recommended the use of the 5 × 5.2 Gy schedule, and underlined its equivalence with the 15 fractions schedule. Its use was further endorsed at a meeting in April 2021. Additionally, we saw quite a large variation in the use of 5 fractions between the different types of radiotherapy institutes. Factors associated with the chance of receiving 5 fractions of 5.2 Gy were age, tumour size, (y)pN, partial breast irradiation (PBI), and type of radiotherapy institute.

This study aimed to provide insights in the use of ultra-hypofractionated radiotherapy during 2020–2023, by analysing both registry and questionnaire data. Specifically, we investigated trends and variation in utilisation, identified factors associated with its use, and identified facilitators and barriers for its implementation. These findings may thereby help to reduce variation across patients and radiotherapy institutes.

## Methods

This study used both registry and questionnaire data. Data of the Netherlands Cancer Registry (NCR) and the NABON Breast Cancer Audit-Radiotherapy (NBCA-R) were used to study 1) trends and variation in use of different radiotherapy schedules over time and, 2) factors associated with the chance of receiving 5 fractions. Questionnaire data was used to identify facilitators and barriers for the implementation of the 5-fraction schedule.

### Registry data

#### Patients

Women eligible for receiving 5 fractions according to the consensus recommendations of the LPRM were included in the current study. Eligible women were aged 40 or older, had a (y)pTis,0-3N0-1M0 tumour after breast conserving surgery or mastectomy without direct reconstruction, and had no indication for lymph node irradiation. Women treated with a boost were included, as the chance of receiving a boost or 5 fractions is very much dependent on each other. Changes in boost indications and shared-decision making during the COVID-19 pandemic could have resulted in women becoming ineligible for a boost and eligible for the 5-fractions schedule [3]. Women with missing pT or pN were included in the dataset if they met the other criteria. Included women were treated with postoperative radiotherapy between January 2020 and December 2023. Women were excluded if they had a history of breast cancer or a synchronous breast tumour (diagnosed within 91 days), or if the dosimetry and/or fractionation schedule was unknown. Presence of comorbidities was no exclusion criteria. Data on patient-, tumour-, treatment-, and radiotherapy institute-related characteristics were obtained from the NCR. These characteristics included amongst others age, income, tumour grade, multifocality, (y)pT, (y)pN, and type of radiotherapy institute. The NCR is a nationwide population-based registry, including records on all newly diagnosed malignancies notified through the Dutch Nationwide Pathology Databank (Palga). Since the NCR does not contain detailed radiotherapy data, these were obtained from the NBCA-R [9]. The NBCA-R includes information on radiotherapy treatment (extracted from the Digital Imaging and Communication in Medicine (DICOM) radiotherapy data), including data on radiotherapy planning CT-scans and dosimetry and fractionation schedules. The NBCA-R was established in 2020. Available data from 17 of the 19 Dutch radiotherapy institutes were linked to the NCR.

### Definitions

Four schedules of radiotherapy were defined: 1) 5 fractions, 2) 15 fractions without boost, 3) 20 fractions with boost (20 fractions with concurrent boost or 15 fractions followed by 5 fractions with a boost), 4) other. Median house hold income per zip code area, provided by Statistics Netherlands (CBS), was used as a proxy for income. Each zip code area covered on average 17 households. Income was divided into lower (< €24.300), intermediate (between €24.300 and €31.000) or higher (≥ €31.000) income. Tumour subtype was classified as hormone (HR) receptor positive (oestrogen and/or progesterone receptor positive) and HER2 + , HR + /HER2-, HR- (oestrogen and progesterone receptor negative) and HER2 + , or HR-/HER2-. Three radiotherapy target volumes were distinguished: whole breast irradiation (WBI), partial breast irradiation (PBI), and post-mastectomy radiotherapy (PMRT). In the Netherlands, radiotherapy care is provided in departments integrated in general or academic hospitals, or in independent radiotherapy institutes. The radiotherapy start date was determined using the NBCA-R. If unavailable, the NCR start date of radiotherapy was used. If both were missing, the median interval between the date of the radiotherapy planning CT-scan and start of radiotherapy of women with known start date of radiotherapy (specific to each institute) were added to the date of the planning CT-scan to estimate the radiotherapy start date.

### Statistical analyses

Trends in the use of the four radiation schedules were visualised. Descriptive statistics were used to describe baseline characteristics. Multiple Imputation by Chained Equations (MICE) was used to impute missing variables needed for analyses (income, tumour grade, multifocality, (y)pT, (y)pN, radiotherapy target volume), using all baseline characteristics. Imputation was repeated 25 times, and the estimates were pooled using Rubin’s rules [13]. For comparison, analyses were performed with both the imputed and complete-case dataset.

Boxplots were constructed to investigate variation between radiotherapy institutes in 5-fraction use, per start year of radiation. To account for variation due to patient- and tumour-related characteristics, a case-mix correction for age, income, tumour grade, multifocality, (y)pT, and (y)pN was performed. Logistic regression was used to test the association between age, income, tumour grade, multifocality, (y)pT, (y)pN, radiotherapy target volume, type of radiotherapy institute, start year of radiation and the chance of receiving 5 fractions, by calculating odds ratios (ORs) and 95% confidence intervals (95%CIs). Logistic regression was used for all start years of radiotherapy combined and for 2020–2022 and 2023 separately. We analysed 2020–2022 and 2023 separately to investigate latest associations and compare this with previous years.

All statistical analyses were performed in Stata Statistical Software (release 17.0, College Station, TX: StataCorp LLC). P-values were two-sided, and a *p*-value < 0.05 was regarded as statistically significant.

### Questionnaires

A questionnaire was developed together with two breast radiation oncologists of the LPRM to get an overview of the use of the 5 × 5.2 Gy schedule amongst radiotherapy institutes in the Netherlands. Questions and answer options were based on the experience of these radiation oncologists, and by the experiences heard in the field. It was always possible to type your own answer if this was not a pre-defined option. The questionnaire was distributed among all 19 radiotherapy institutes in April 2024. Each institute was asked to fill out the questionnaire once. The questions focussed on identifying which patients received the 5 × 5.2 Gy schedule, and the facilitators and barriers for implementing this schedule. We focused on the 5 × 5.2 Gy schedule as previous research showed that the 5 × 5.7 Gy schedule was only barely used at the end of 2021 [7]. Additionally, at the LPRM meeting in November 2020 the 5 × 5.2 Gy schedule was preferred above the 5 × 5.7 Gy schedule. The questionnaire (Additional File 1) consisted of general questions (i.e. radiotherapy target volume), patient-related questions (e.g. age), and non-patient-related questions (e.g. period of implementation of the 5-fraction schedule and financial constraints). The questions regarding radiotherapy target volume focussed on WBI, PBI, PMRT and local radiotherapy + radiotherapy to axillary lymph node levels 1–2. Local radiotherapy was defined as WBI after breast-conserving surgery, or PMRT after mastectomy. The response to the questions were individually analysed and the results were discussed descriptively or visualised using bar graphs.

## Results

### Registry data

#### Baseline characteristics

A total of 25,644 women were eligible for inclusion in the current study (Additional File 2, Fig. 1), of which 16,119 were included in the analyses. Between January 2020 and December 2023, a total of 6,483 women (40.2%) received 5 fractions, 6,297 received 15 fractions (39.1%), 3,229 received 20 fractions (20.0%) and 106 received another radiation schedule (0.7%) (Table 1). Additional File 2, Table 1 includes details of the received fractions and doses per radiation group. Compared to women receiving 15 or 20 fractions, those receiving 5 fractions were less often aged 40—49 (5 fractions: 4.7%, 15 fractions: 16.3%, 20 fractions: 19.9%), had more often a screen-detected tumour (59.6% vs 47.9% and 46.3%, respectively), received more often PBI (39.2% vs 9.9% and 0.1%, respectively), and were more often treated in an academic radiotherapy institute (51.0% vs 34.2% and 40.9%, respectively).

**Fig. 1:**
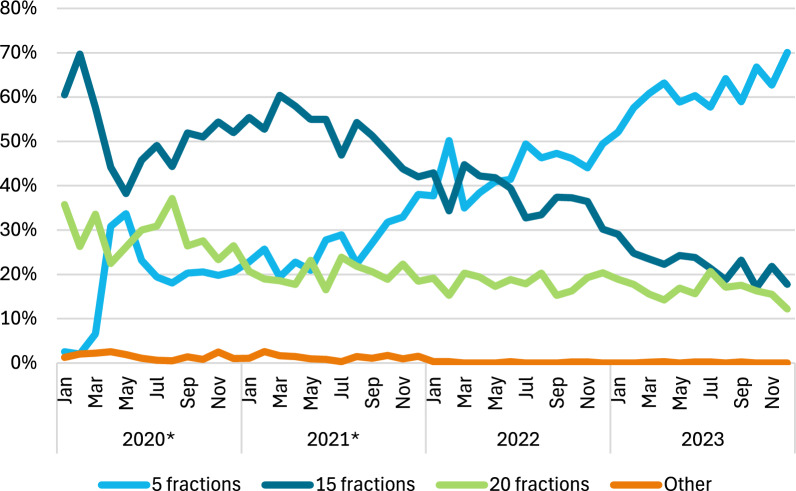
Radiotherapy schedules of patients treated with radiotherapy in the Netherlands from 2020 until 2023. LPRM: National Platform Radiotherapy Breast Cancer. *: March 2020 – Start COVID-19 pandemic and publication of COVID-related measures, April and July 2020 – Publication FAST trials, November 2020 and April 2021: LPRM meetings

**Table 1:** Baseline characteristics of the included population

	Total	5 fractions	15 fractions	20 fractions	Other
Total	16,115	6,483	6,297	3,229	106
*Age*					
40–49	1,977 (12.3)	302 (4.7)	1,029 (16.3)	641 (19.9)	5 (4.7)
50–75	12,913 (80.1)	5,545 (85.5)	4,888 (77.6)	2,393 (74.1)	87 (82.1)
> 75	1,225 (7.6)	636 (9.8)	380 (6.0)	195 (6.0)	14 (13.2)
*Income*					
Low	3,520 (21.8)	1,445 (22.3)	1,362 (21.6)	690 (21.4)	23 (21.7)
Middle	5,841 (36.2)	2,241 (34.6)	2,354 (37.4)	1,196 (37.0)	50 (47.2)
High	6,114 (37.9)	2,475 (38.2)	2,400 (38.1)	1,207 (37.4)	32 (30.2)
Missing	640 (4.0)	322 (5.0)	181 (2.9)	136 (4.2)	1 (0.9)
*Detected by screening*					
No	7,626 (47.3)	2,589 (39.9)	3,267 (51.9)	1,728 (53.5)	42 (39.6)
Yes	8,440 (52.4)	3,865 (59.6)	3,017 (47.9)	1,494 (46.3)	64 (60.4)
Missing	49 (0.3)	29 (0.4)	13 (0.2)	7 (0.2)	0 (0.0)
*Lateralisation*					
Left	8,163 (50.7)	3,256 (50.2)	3,208 (50.9)	1,641 (50.8)	58 (54.7)
Right	7,952 (49.3)	3,227 (49.8)	3,089 (49.1)	1,588 (49.2)	48 (45.3)
*Histology*					
Ductal	13,105 (81.3)	5,262 (81.2)	4,992 (79.3)	2,764 (85.6)	87 (82.1)
Lobular/mixed	2,023 (12.6)	757 (11.7)	971 (15.4)	285 (8.8)	10 (9.4)
Other	987 (6.1)	464 (7.2)	334 (5.3)	180 (5.6)	9 (8.5)
*Tumour grade*					
1	3,735 (23.2)	1,897 (29.3)	1,475 (23.4)	324 (10.0)	39 (36.8)
2	7,898 (49.0)	3,312 (51.1)	3,295 (52.3)	1,234 (38.2)	57 (53.8)
3	4,145 (25.7)	1,166 (18.0)	1,363 (21.6)	1,608 (49.8)	8 (7.5)
Missing	337 (2.1)	108 (1.7)	164 (2.6)	63 (2.0)	2 (1.9)
*Multifocality*					
No	14,600 (90.6)	6,041 (93.2)	5,556 (88.2)	2,902 (89.9)	101 (95.3)
Yes	1,461 (9.1)	417 (6.4)	728 (11.6)	311 (9.6)	5 (4.7)
Missing	54 (0.3)	25 (0.4)	13 (0.2)	16 (0.5)	0 (0.0)
*Subtype*					
HR + /HER2 +	1,109 (6.9)	329 (5.1)	496 (7.9)	278 (8.6)	6 (5.7)
HR + /HER2-	10,882 (67.5)	4,859 (74.9)	4,293 (68.2)	1,642 (50.9)	88 (83.0)
HR-/HER2 +	552 (3.4)	167 (2.6)	292 (4.6)	92 (2.8)	1 (0.9)
HR-/HER2-	1,600 (9.9)	429 (6.6)	475 (7.5)	692 (21.4)	4 (3.8)
Missing	1,972 (12.2)	699 (10.8)	741 (11.8)	525 (16.3)	7 (6.6)
*DCIS component*					
No	7,546 (46.8)	3,166 (48.8)	3,108 (49.4)	1,216 (37.7)	56 (52.8)
Yes	6,814 (42.3)	2,687 (41.4)	2,552 (40.5)	1,531 (47.4)	44 (41.5)
DCIS	1,299 (8.1)	381 (5.9)	539 (8.6)	373 (11.6)	6 (5.7)
Missing	456 (2.8)	249 (3.8)	98 (1.6)	109 (3.4)	0 (0.0)
*(y)pT*					
is	1,649 (10.2)	545 (8.4)	648 (10.3)	450 (13.9)	6 (5.7)
0^a^	1,127 (7.0)	367 (5.7)	671 (10.7)	88 (2.7)	1 (0.9)
1	10,330 (64.1)	4,591 (70.8)	3,676 (58.4)	1,974 (61.1)	89 (84.0)
2	2,591 (16.1)	807 (12.4)	1,157 (18.4)	619 (19.2)	8 (7.5)
3	140 (0.9)	34 (0.5)	76 (1.2)	28 (0.9)	2 (1.9)
Missing	278 (1.7)	139 (2.1)	69 (1.1)	70 (2.2)	0 (0.0)
*(y)pN*					
0	14,200 (88.1)	5,830 (89.9)	5,403 (85.8)	2,867 (88.8)	100 (94.3)
1	919 (5.7)	203 (3.1)	575 (9.1)	137 (4.2)	4 (3.8)
Missing	996 (6.2)	450 (6.9)	319 (5.1)	225 (7.0)	2 (1.9)
*Neo-adjuvant systemic therapy*					
No	12,942 (80.3)	5,561 (85.8)	4,946 (78.5)	2,341 (72.5)	94 (88.7)
Yes	3,173 (19.7)	922 (14.2)	1,351 (21.5)	888 (27.5)	12 (11.3)
*RT target volume*					
WBI	12,111 (75.2)	3,528 (54.4)	5,366 (85.2)	3,190 (98.8)	27 (25.5)
PMRT	326 (2.0)	60 (0.9)	237 (3.8)	25 (0.8)	4 (3.8)
PBI	3,248 (20.2)	2,544 (39.2)	625 (9.9)	4 (0.1)	75 (70.8)
Missing	430 (2.7)	351 (5.4)	69 (1.1)	10 (0.3)	0 (0.0)
*Type of RT institute*^*b*^					
Independent	5,308	1,895 (35.7)	2,444 (46.0)	959 (18.1)	10 (0.2)
General	4,018	1,279 (31.8)	1,699 (42.3)	950 (23.6)	90 (2.2)
Academic	6,789	3,309 (48.7)	2,154 (31.7)	1,320 (19.4)	6 (0.1)
*Start year of RT*^*b*^					
2020	2,843 (17.6)	525 (8.1)	1,464 (23.2)	810 (25.1)	44 (41.5)
2021	4,060 (25.2)	1,086 (16.8)	2,107 (33.5)	815 (25.2)	52 (49.1)
2022	4,300 (26.7)	1,887 (29.1)	1,620 (25.7)	788 (24.4)	5 (4.7)
2023	4,912 (30.5)	2,985 (46.0)	1,106 (17.6)	816 (25.3)	5 (4.7)

HR: hormone receptor, PBI: partial breast irradiation, PMRT: post-mastectomy radiotherapy, RT: radiotherapy, WBI: whole breast irradiation

^a^(y)pT can be 0 after neo-adjuvant therapy and a pathological complete response, or when the tumour is only found in the lymph nodes

^b^Percentages add up vertically to 100%

### Trends in the use of 5 fractions

**Fig. 2:**
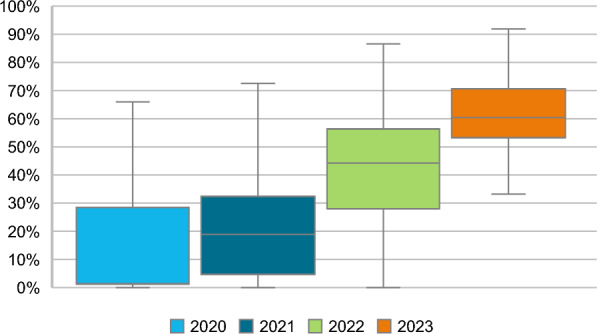
Case-mix adjusted variation between radiotherapy institutes in the percentage of patients treated with 5 fractions Logistic regression

The percentages of women treated with 5 fractions increased over the years, with 18.5% of eligible women receiving 5 fractions in 2020 and 60.8% in 2023 (Fig. 1). The percentages of women treated with 15 or 20 fractions decreased from 51.5 to 22.5% and from 28.5 to 16.6%, respectively, between 2020 and 2023. After case-mix correction, and by using the imputed dataset, the lowest variation between radiotherapy institutes in the percentage of patients treated with 5 fractions was seen in 2023, with a median percentage of patients treated with 5 fractions of 60.4% (interquartile range: 53.3–70.6%) (Fig. 2). Additional File 2, Table 2 shows the results without case-mix correction and in the complete case dataset.

Multivariable logistic regression analysis showed a higher chance for receiving 5 fractions for women aged > 75 vs 50–75 (OR: 2.13), those with (y)pT0 vs (y)pT1 tumours (OR: 1.59), PBI vs WBI (OR: 8.40), treatment at academic vs independent radiotherapy institutes (OR: 2.64), and those starting radiotherapy in 2021, 2022, or 2023 vs 2020 (OR: 1.54, 3.89, 10.18, respectively) (Table 2). A lower chance of receiving 5 fractions was seen for women aged 40–49 vs 50–75 (OR: 0.28), those with grade 3 vs grade 1 tumours (OR: 0.71), multifocal vs unifocal tumours (OR: 0.75), (y)pT2 vs (y)pT1 tumours (OR: 0.84), (y)pN1 vs (y)pN0 tumours (OR: 0.65), PMRT vs WBI (OR: 0.68), and treatment at general vs independent radiotherapy institutes (OR: 0.88). When comparing the adjusted results of 2020–2022 with those of 2023 no differences were seen between the two periods, except for the target volume. In 2023, a lower chance for receiving 5 fractions was seen for PMRT vs WBI (OR: 0.53), but not for 2020–2022 (OR: 0.74). Results of the complete case-analyses are shown in Additional File 2 – Table 3.

**Table 2:** Association between various characteristics and the chance of receiving 5 fractions

	2020–2023	2020–2022		2023		
	Unadjusted OR, 95% CI	Adjusted OR, 95% CI^a^	Unadjusted OR, 95% CI	Adjusted OR, 95% CI^a^	Unadjusted OR, 95% CI	Adjusted OR, 95% CI^a^
*Age*						
40–49	**0.24 (0.21–0.27)**	**0.28 (0.24–0.32)**	**0.35 (0.30–0.95)**	**0.47 (0.40–0.56)**	**0.10 (0.08–0.13)**	**0.13 (0.10–0.16)**
50–75	Reference	Reference	Reference	Reference	Reference	Reference
> 75	**1.43 (1.28–1.61)**	**2.13 (1.85–2.46)**	**1.31 (1.13–1.52)**	**2.02 (1.69–2.42)**	**1.68 (1.33–2.12)**	**2.45 (1.89–3.16)**
*Income*						
Low	Reference	Reference	Reference	Reference	Reference	Reference
Middle	**0.90 (0.82–0.98)**	0.95 (0.86–1.06)	**0.86 (0.77–0.95)**	0.98 (0.86–1.11)	0.94 (0.80–1.10)	0.92 (0.76–1.11)
High	0.98 (0.90–1.06)	0.91 (0.82–1.01)	0.99 (0.90–1.10)	0.93 (0.82–1.06)	0.90 (0.77–1.06)	0.87 (0.72–1.05)
*Tumour grade*						
1	Reference	Reference	Reference	Reference	Reference	Reference
2	**0.69 (0.64–0.75)**	0.96 (0.87–1.07)	**0.69 (0.63–0.76)**	1.03 (0.91–1.16)	**0.61 (0.52–0.71)**	0.86 (0.71–1.03)
3	**0.38 (0.34–0.41)**	**0.71 (0.62–0.80)**	**0.39 (0.34–0.44)**	**0.86 (0.73–1.00)**	**0.30 (0.25–0.35)**	**0.51 (0.41–0.63)**
*Multifocality*						
No	Reference	Reference	Reference	Reference	Reference	Reference
Yes	**0.57 (0.50–0.64)**	**0.75 (0.65–0.86)**	**0.50 (0.42–0.59)**	**0.73 (0.61–0.88)**	**0.52 (0.43–0.63)**	**0.80 (0.65–0.99)**
*(y)pTb*						
is	**0.62 (0.56–0.69)**	0.96 (0.83–1.10)	**0.64 (0.56–0.73)**	0.87 (0.73–1.02)	**0.69 (0.55–0.85)**	1.17 (0.90–1.51)
0	**0.61 (0.53–0.69)**	**1.59 (1.36–1.86)**	**0.62 (0.53–0.73)**	**1.42 (1.18–1.71)**	**0.70 (0.54–0.90)**	**1.99 (1.47–2.69)**
1	Reference	Reference	Reference	Reference	Reference	Reference
2	**0.57 (0.52–0.63)**	**0.84 (0.75–0.94)**	**0.52 (0.46–0.59)**	**0.86 (0.74–0.99)**	**0.57 (0.49–0.67)**	**0.79 (0.66–0.95)**
3	**0.41 (0.28–0.61)**	0.95 (0.59–1.54)	**0.51 (0.32–0.82)**	1.28 (0.72–2.25)	**0.26 (0.13–0.53)**	0.59 (0.26–1.35)
*(y)pN*						
0	Reference	Reference	Reference	Reference	Reference	Reference
1	**0.42 (0.36–0.50)**	**0.65 (0.54–0.78)**	**0.39 (0.32–0.49)**	**0.62 (0.49–0.78)**	**0.47 (0.36–0.61)**	**0.68 (0.51–0.92)**
*RT target volume*						
WBI	Reference	Reference	Reference	Reference	Reference	Reference
PMRT	**0.57 (0.43–0.76)**	**0.68 (0.48–0.96)**	0.72 (0.51–1.01)	0.74 (0.49–1.12)	**0.42 (0.25–0.71)**	**0.53 (0.29–0.99)**
PBI	**8.44 (7.70–9.25)**	**8.40 (7.52–9.39)**	**8.53 (7.69–9.48)**	**8.48 (7.46–9.63)**	**18.79 (14.11–25.03)**	**13.20 (9.79–17.79)**
*Type of RT institute*						
Independent	Reference	Reference	Reference	Reference	Reference	Reference
General	**0.84 (0.77–0.92)**	**0.88 (0.79–0.98)**	1.06 (0.94–1.19)	0.92 (0.80–1.05)	0.93 (0.80–1.08)	0.95 (0.80–1.13)
Academic	**1.71 (1.59–1.84)**	**2.64 (2.40–2.89)**	**2.73 (2.47–3.01)**	**3.49 (3.10–3.93)**	**1.37 (1.20–1.56)**	**1.57 (1.34–1.83)**
*Start year of RT*						
2020	Reference	Reference	Reference	Reference		
2021	**1.61 (1.43–1.81)**	**1.54 (1.35–1.76)**	1.61 (1.43–1.81)	1.57 (1.37–1.79)		
2022	**3.45 (3.09–3.86)**	**3.89 (3.42–4.43)**	3.45 (3.09–3.86)	3.96 (3.48–4.51)		
2023	**6.89 (6.12–7.64)**	**10.18 (8.94–11.58)**				

CI: confidence interval, OR: odds ratio, PBI: partial breast irradiation, PMRT: post-mastectomy radiotherapy, RT: radiotherapy, WBI: whole breast irradiation

Analyses were performed for 2020–2023, 2020–2022 and 2023, using the imputed dataset

^a^Adjusted for all variables mentioned in the table

^b^(y)pT can be 0 after neo-adjuvant therapy and a pathological complete response, or when the tumour is only found in the lymph nodes

### Questionnaire

**Fig. 3:**
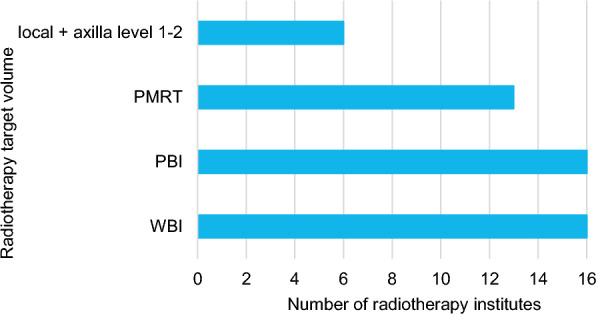
Number of radiotherapy institutes who provided 5 × 5.2 Gy in April 2024, per radiotherapy target volume. PBI: partial breast irradiation, PMRT: post-mastectomy radiotherapy, WBI: whole breast irradiation

**Fig. 4:**
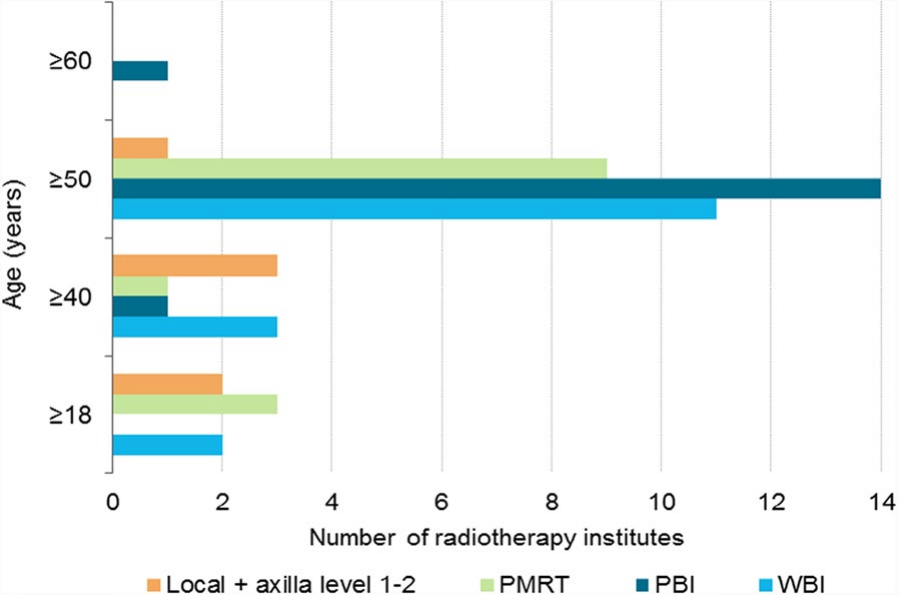
Ages at which 5 × 5.2 Gy was provided in April 2024, per radiotherapy target volume. PBI: partial breast irradiation, PMRT: post-mastectomy radiotherapy, WBI: whole breast irradiation

**Fig. 5:**
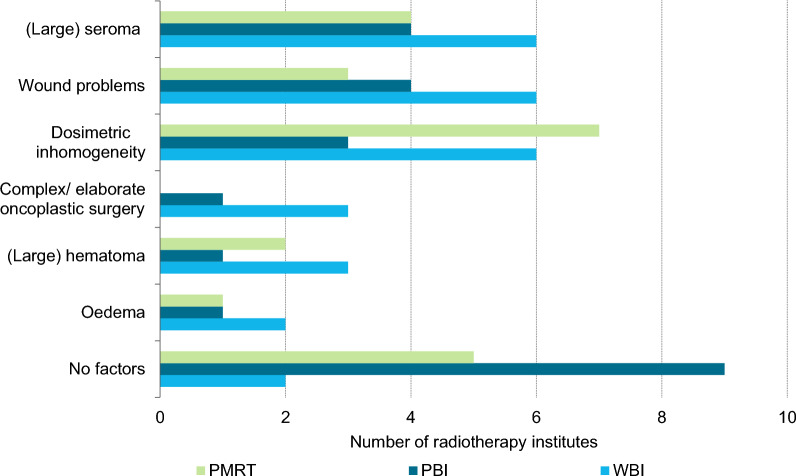
Reasons to refrain from using 5 × 5.2 Gy in April 2024, by radiotherapy target volume. PBI: partial breast irradiation, PMRT: post-mastectomy radiotherapy, WBI: whole breast irradiation

**Fig. 6:**
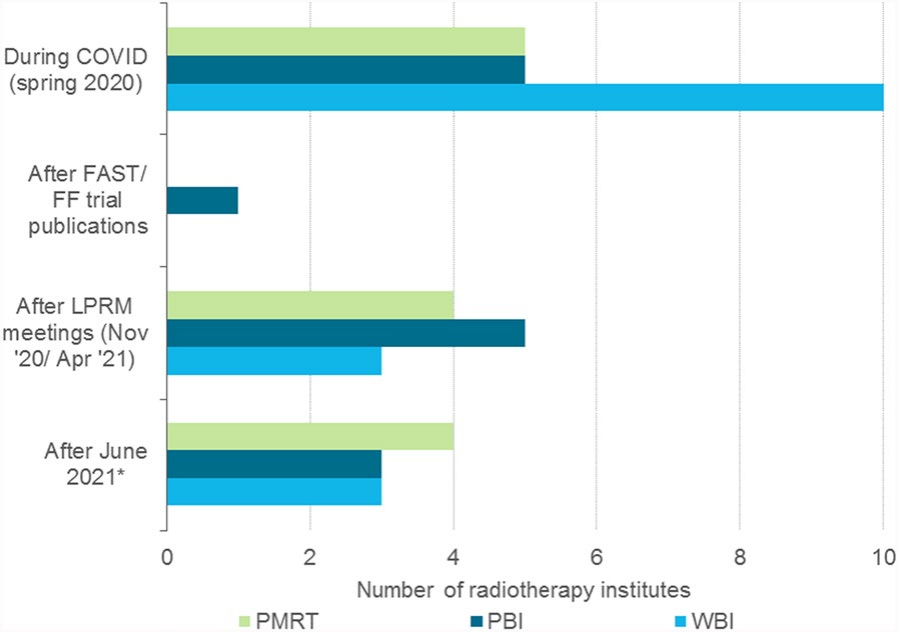
First implementation of 5 × 5.2 Gy and/or 5 × 5.7 Gy over time. PBI: partial breast irradiation, PMRT: post-mastectomy radiotherapy, RT: radiotherapy, WBI: whole breast irradiation. *LPRM guideline was updated in June 2021

A total of 16 out of 19 RT institutes responded to the questionnaire (84.2%). All responding institutes used the 5 × 5.2 Gy schedule for both WBI and PBI, and most for PMRT. Additionally, some applied the 5 × 5.2 Gy schedule for local radiotherapy + radiotherapy to the axillary lymph node levels 1–2 (Fig. 3). Most institutes implemented the 5 × 5.2 Gy schedule in patients aged ≥ 50 (Fig. 4). Some institutes already implemented the schedule in patients aged ≥ 18 or ≥ 40, while one institute only implemented this schedule in patients aged ≥ 60. The majority of the institutes mentioned no standard factors for refraining from using the 5 × 5.2 Gy schedule (Fig. 5). At least 3 institutes mentioned they refrained from using the 5 × 5.2 Gy schedule because of a (large) seroma, wound problems or dosimetric inhomogeneity. This accounted for all target volumes. Factors only mentioned once can be found in Additional File 2 – Table 4. Two institutes reported that the implementation of the 5 × 5.2 Gy schedule was delayed due to financing/reimbursement of the 5-fraction schedules. The majority of institutes reported that the 5-fraction schedules for WBI were implemented for the first time during the COVID-19 pandemic (Fig. 6). The scheme was implemented to reduce the pressure on the healthcare sector (N = 9) and to prevent further infections with SARS-COV-2 (N = 9). Additionally, the LPRM consensus meeting in November 2020 and April 2021 influenced the use of the 5-fraction schedule.

## Discussion

The current study showed an increase in the use of the 5-fraction schedule from 2020 to 2023 in women aged 40 years or older, diagnosed with a (y)pTis,0-3N0-1M0 tumour after breast-conserving surgery or mastectomy without direct reconstruction, without lymph node irradiation. Variation between radiotherapy institutes in the use of this schedule decreased over the years. Registry data showed an association between age, tumour grade, multifocality, (y)pT, (y)pN, radiotherapy target volume, type of radiotherapy institute, the start year of radiotherapy and the use of 5 fractions. Results of the questionnaire showed variation between radiotherapy institutes in the implementation of the 5-fraction schedule regarding age, radiotherapy target volume, and time of implementation. Most institutes mentioned no barriers for using 5 fractions.

According to the registry data and the questionnaire, the start of the COVID-19 period coincided with an increase in the use of the 5-fraction schedule. This change was also described in other countries [11, 14]. In March 2020, COVID-19 related international and national radiotherapy guidelines were formulated, recommending the use of the 5-fraction schedule [3]. These guidelines probably stimulated its use. According to both the registry and questionnaire data, the publication of the FAST and FAST-Forward trial itself was not an important reason to implement the 5-fraction schedule. The LPRM meetings of November 2020 and April 2021 were important events for a broader implementation of the 5-fraction schedule. A further increase in the use of 5 fractions in 2022 and 2023 was observed in our study. However, some institutes still experienced barriers for its implementation, as shown by the questionnaire, such as (large) seroma, wound problems and dosimetric inhomogeneity. These results suggest that although the LPRM stimulated the use of five fractions, experience in the use of a new treatment is very important for the acceptance of trial results and full implementation of new treatments, and this takes time.

Our results showed a decrease in the use of a radiotherapy boost in 2021–2023. In the FAST-Forward trial patients were allowed to receive a sequential boost after receiving 5 fractions of radiotherapy [2]. In the Netherlands, however, a boost is almost always given simultaneously integrated. Hence, Dutch patients treated with a boost were not eligible for 5 fractions. According to the registry data 39 out of the 6,483 patients treated with 5 fractions received a simultaneous boost (0.6%). This was not a specific patient group. At the LPRM meeting in November 2020 the guideline, when to give a boost, was amended. Giving a boost should more often be decided by shared decision making. The resulting decrease in the use of a boost allowed for a further increase in the use of the 5-fraction schedule. Women treated with a boost were therefore also included in the current study, as the chance of receiving a boost or 5 fractions is very much dependent on each other.

Although variation between radiotherapy institutes decreased, considerable variation remained in 2023. We showed that this was mostly based on patient age or factors associated with toxicity (seroma, wound problems, dosimetric inhomogeneity). Additionally, two radiotherapy institutions reported that the implementation of the 5 × 5.2 Gy schedule was delayed due to financing/reimbursement, even though in the Netherlands the reimbursement for 15 and 5 fractions is generally comparable. However, at the introduction of the breath-hold technique a higher reimbursement could be declared for left-sided radiotherapy in combination with a breath-hold technique, provided patients received at least six fractions. Currently, reimbursement for radiotherapy, with or without a breath-hold technique, is generally comparable across institutions. The main advantage of the 5-fraction is that it limits the burden on the patient and health care resources. Especially academic hospitals had a shortage in lab technicians, possibly explaining why they were early adaptors of the 5 fraction schedule. Meetings with representatives of all radiotherapy institutes, to discuss the findings of such a questionnaire, are highly recommended to decrease variation between departments. During these meetings radiotherapy institutes can learn from each other.

Comparable to our results, a previous study, including data till May 2021, showed a decreased chance of receiving 5 fractions for patients aged 18–49 and for those with higher stage tumours [4]. A lower chance of receiving 5 fractions for younger patients can be expected, as in the Dutch guidelines shared-decision making can be considered in patients aged 40–49 concerning the use of 5 fractions, as only the 5 year follow-up results are known. The questionnaire data showed that 3 institutes provided 5 fractions to patients aged 18–40 years. According to the guidelines, those patients are not eligible for 5 fractions, and hence they were not included in the analyses. Examination of the crude registry data showed that in total 22 patients aged 18–40 years received 5 fractions during 2020–2023. The questionnaire was filled out in April 2024, while the data was collected until December 2023. Over time radiotherapy institutions may be increasingly adopting a broader approach of the inclusion criteria. According to the registry data, patients aged > 75 were more likely to receive 5 fractions. This was because at the introduction of the 5-fraction schedule most institutes only gave 5 fractions to older ages.

Both our registry and questionnaire data showed a lower chance of receiving 5 fractions for those receiving PMRT, and for those receiving radiotherapy to the axillary lymph node levels 1–2. This could be due to the small number of patients in the FAST-Forward study with PMRT and 5 × 5.2 Gy (N = 84), and the exclusion of patients needing radiotherapy to any regional lymph nodes. This implies limited level of evidence, which might have been taken into account in the daily practice. Currently, a sub study of the FAST-Forward trial investigates the effect of ultra-hypofractionation on the lymph nodes. Furthermore, in a previous study of our group, including data from 2020–2021, we found an association between age, (y)pN0, radiotherapy target volume, and type of radiotherapy institute and the chance of receiving 5 fractions for patients eligible for 5 × 5.2 Gy. An association with tumour size was only found in 2021. Beside those factors, the current study also showed an association for tumour grade and multifocality. (y)pT, (y)pN, and tumour grade all indicate a lower risk of recurrence [20].

Strengths of this study include the inclusion of registry data from 17 out of 19 radiotherapy institutes in the Netherlands, thereby covering around 85% of the patients treated in the Netherlands. Additionally, the use of a questionnaire was used to support the registry data and vice versa. A limitation is that the NBCA-R first started registering patients in 2020. In this year not all radiotherapy institutes provided their data to the NBCA-R, where academic institutes reported a higher percentage of their patient population in the NBCA-R as compared to general and independent institutes. Patients treated in academic radiotherapy institutes were more likely to receive 5 fractions. Consequently, the trendline for the use of the 5-fraction schedule (Fig. 1) would most likely have been lower when the data of all other radiotherapy institutes would have been included at the start as well. However, it is not expected that this influenced the other study results. Additionally, the NCR does not include data on comorbidities, meaning we could not investigate the association between comorbidities and the chance of receiving 5 fractions. Furthermore, the questionnaire was anonymous, meaning we could not contact the relevant radiotherapy institutes to discuss why their use of the 5-fraction schedules differed from others.

## Conclusions

The use of ultra-hypofractionated radiotherapy for breast cancer has increased during the past four years in the Netherlands, with reduced variation across institutes. The COVID-19 pandemic coincided with the first implementation of the 5-fraction schedule. National meetings appeared instrumental in driving implementation. Additionally, our results showed important factors determining the chance of receiving 5 fractions. Even in 2023 the data showed that not all patients eligible for this schedule actually received it. Variation between institutes in the implementation of the schedule can be caused by factors such as age, radiotherapy target volume, and fear of toxicity.

## Supplementary Information

Additional file 1

Additional file 2

## Data Availability

The dataset supporting the conclusion of this article was used under license, and so are not publicly available. Data are available via the NCR and NBCA-R upon request and after approval by the NCR and NBCA-R. The plan for the statistical analysis will be made available by the corresponding author upon request.

## Abbreviations

HR: Hormone receptor
LPRM: National Platform Radiotherapy Breast Cancer
NCR: Netherlands cancer registry
NBCA-R: NABON Breast Cancer Audit-Radiotherapy
PBI: Partial breast irradiation
PMRT: Post-mastectomy radiotherapy
WBI: Whole-breast irradiation
