# Supplementary material for: Implementation of ultra-hypofractionated radiotherapy for breast cancer in the Netherlands in 2020–2023, using registry data and questionnaires
Source: Radiat Oncol. 2025 Jun 12;20:99. doi: 10.1186/s13014-025-02669-w (PMC12164123; doi:10.1186/s13014-025-02669-w)
Supplement: Supplementary file 1 — Additional file 1 [file 13014_2025_2669_MOESM1_ESM.docx]

Supplementary Method

Remarks about the questionnaire:

The following questions were translated from the Dutch version of the questionnaire that was distributed.

⊗This sign is visible in multiple choice questions. When this answer option is selected, all other multiple choice answer options in that question are deselected.

Square answer options mean it is a multiple choice question, round answer options are single choice questions.

## Ultra-hypofractionation questions

General introduction: This is an anonymous questionnaire about ultra-hypofractionated radiotherapy schedules among breast cancer patients. The questionnaire focusses on the 5x5.2 Gy arm from the FAST-Forward trials.

Please answer 1 questionnaire per institute.

Expected time is 10-15 minutes.

Q1

Which of the following radiotherapy target volume do you use the 5x5.2 Gy schedule for? (Multiple answers possible)

- Whole Breast Irradiation (WBI)
- Partial Breast Irradiation (PBI)
- Thoracic wall irradiation
- Local + axilla levels 1-2
- Local + axilla levels 1/2 to 4
- Local + axilla level 1/2 to 4 and parasternal
- Axilla levels 1-2
- Axilla level 1/2 to 4

⊗ None of the above, namely

The following questions focus on factors of influence on the decision to irradiate or not with 5x5.2 Gy.

Q2

Show question if:

Q1 is answered with: Whole Breast irradiation (WBI)

What factors contribute to the decision to irradiate using the 5x5.2 Gy schedule with **WBI**? (multiple answers possible)

- Cup size, namely... (see follow-up question on next page)
- Volume
- Cross-section
- Inhomogeneity
- Other, namely

⊗ None of these factors influence the decision

Q3

Show question if:

Q2 is answered with: Cupsize, namely… (see follow-up question on next page)

What cup sizes contribute to the decision to irradiate with the 5x5.2 Gy schedule with WBI? (follow-up question)

- From cup size D
- From cup size E
- From cup size F
- From cup size G
- Other, namely

Q4

Show question if:

Q1 is answered with: Whole Breast Irradiation(WBI)

In case of **WBI**, does the use of 5x5.2 Gy depend on age?

- Yes, from the age of 40
- Yes, from the age of 50
- Yes, from the age of 60
- Yes, from the age of 70
- No
- Other, namely

Q5

Show question if:

Q1 is answered with: Local + axilla levels 1-2

Or Local + axilla levels 1/2 to 4

In case of **local + axilla levels 1/2 (to 4)** as radiotherapy target volume, does the use of 5x5.2 Gy depend on age?

- Yes, from the age of 40
- Yes, from the age of 50
- Yes, from the age of 60
- Yes, from the age of 70
- No
- Other, namely

Q6

Show question if:

Q1 is answered with: Thoraric wall irradiation

What factors contribute to the decision to irradiate using the 5x5.2 Gy schedule with **thoracic wall irradiation**? (multiple answers possible)

- Cross-section
- Inhomogeneity
- Other, namely

⊗ None of these factors influence the decision

Q7

Show question if:

Q1 is answered with: Thoraric wall irradiation

In case of thoracic wall irradiation, does the use of 5x5.2 Gy depend on age?

- Yes, from the age of 18
- Yes, from the age of 30
- Yes, from the age of 40
- Yes, from the age of 50
- Yes, from the age of 60
- Yes, from the age of 70
- No
- Other, namely

Q8

Show question if:

Q1 is answered with: Partial Breast Irradiation (PBI)

What factors contribute to the decision to irradiate using the 5x5.2 Gy schedule with **PBI**? (multiple answers possible)

- Cup size, namely... (see follow-up question on next page)
- Volume
- Cross-section
- Inhomogeneity
- Other, namely

⊗ None of these factors influence the decision

Q9

Show question if:

Q8 is answered with: Cup size, namely… (see follow-up question on next page)

What cup sizes contribute to the decision to irradiate with the 5x5.2 Gy schedule with **PBI**? (follow-up question)

- From cup size D
- From cup size E
- From cup size F
- From cup size G
- Other, namely

Q10

Show question if:

Q1 is answered with: Partial breast irradiation (PBI)

In case of **PBI**, does the use of 5x5.2 Gy depend on age?

- Yes, from the age of 40
- Yes, from the age of 50
- Yes, from the age of 60
- Yes, from the age of 70
- No
- Other, namely

Q11

Are there other **patient-related** reasons not to implement 5x5.2 Gy?

Q12

Does the reimbursement of the ultra-hypofractionated schedule influence the implementation of the schedule?

- Yes, because of this it was not implemented
- Yes, this delayed the implementation
- There was limited implementation (e.g. only PBI), namely
- No

Q13

Are there other non-**patient-related** reasons to implement 5x5.2 Gy?

Q14

Show question if:

Q1 is answered with: Whole Breast Irradiation (WBI)

What was the first time you implemented the ultra-hypofractionated schedule in case of **WBI**?

- During the COVID pandemic (spring 2020)
- After the publications of the FAST-Forward and FAST trials (summer 2020)
- After the LPRM meetings (November 2020/ April 2021)
- After June 2021
- Not implemented
- Unkown
- Other, namely ...

Q15

Show question if:

Q1 is answered with: Partial Breast Irradiation (PBI)

What was the first time you implemented the ultra-hypofractionated schedule in case of **PBI**?

- During the COVID pandemic (spring 2020)
- After the publications of the FAST-Forward and FAST trials (summer 2020)
- After the LPRM meetings (November 2020/ April 2021)
- After June 2021
- Not implemented
- Unkown
- Other, namely ...

Q16

Show question if:

Q1 is answered with: Thoraric wall irradiation

What was the first time you implemented the ultra-hypofractionated schedule in case of **thoracic wall irradiation**?

- During the COVID pandemic (spring 2020)
- After the publications of the FAST-Forward and FAST trials (summer 2020)
- After the LPRM meetings (November 2020/ April 2021)
- After June 2021
- Not implemented
- Unkown
- Other, namely ...

Q17

Did the COVID-19 pandemic contributed to the implementation of the ultra-hypofractionated schedule? If so, in what way? (multiple answers are possible)

- Yes, it was implemented to reduce the pressure on the healthcare sector
- Yes, it was implemented to reduce the chance of COVID-19 infections
- Yes, the implementation of the schedule was delayed because of the unknown toxicity in the start (before the publications of the follow ups of the FAST and FAST-Forward trials)
- Yes, the implementation of the schedule was delayed because of the pressure on the healthcare sector

⊗ No, the COVID-19 pandemic did not contribute to the implementation.

- Other, namely ...
