# Supplementary material for: Implementation of ultra-hypofractionated radiotherapy for breast cancer in the Netherlands in 2020–2023, using registry data and questionnaires
Source: Radiat Oncol. 2025 Jun 12;20:99. doi: 10.1186/s13014-025-02669-w (PMC12164123; doi:10.1186/s13014-025-02669-w)
Supplement: Supplementary file 2 — Additional file 2 [file 13014_2025_2669_MOESM2_ESM.docx]

**
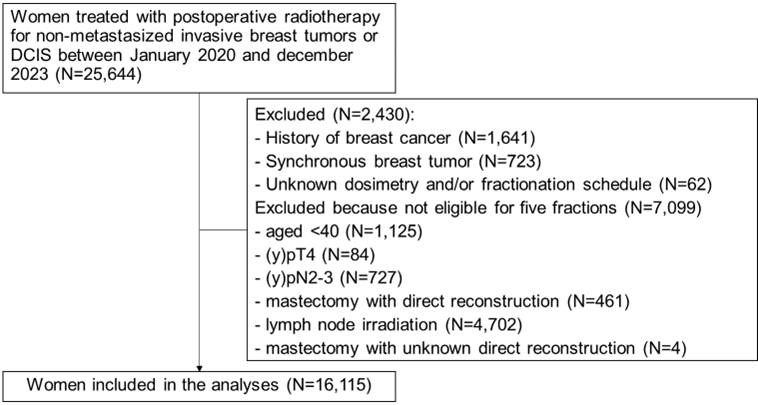
**

**Supplementary Fig. 1** Flowchart of included women.

**Supplementary Table 1** Received fractions and doses per radiation group.

| Fractions | Doses | Frequency | Group |
| --- | --- | --- | --- |
| 5 | 5 | 21 | 5 fractions |
| 5 | 5.2 | 6,013 | 5 fractions |
| 5 | 5.4 | 42 | 5 fractions |
| 5 | 5.7 | 334 | 5 fractions |
| 5 | 6 | 73 | 5 fractions |
| 15 | 2.67 | 6,297 | 15 fractions |
| 20 | 2.18 | 3,125 | 20 fractions |
| 15+5 | 2.67 | 104 | 20 fractions |
| 2 | 2.67 | 1 | Other |
| 4 | 5.2 | 1 | Other |
| 10 | 2.67 | 2 | Other |
| 10 | 3.85 | 78 | Other |
| 10 | 5.2 | 1 | Other |
| 11 | 2.67 | 1 | Other |
| 15 | 5.2 | 1 | Other |
| 17 | 2.18 | 1 | Other |
| 18 | 2.18 | 1 | Other |
| 19 | 2.18 | 1 | Other |
| 20 | 2.5 | 1 | Other |
| 22 | 2.03 | 17 | Other |

**Supplementary Table 2** Variation between radiotherapy institutions in the percentage of patients treated with 5 fractions, using the complete case dataset

|  | 2020, median (IQR) | 2021, median (IQR) | 2022, median (IQR) | 2023, median (IQR) |
| --- | --- | --- | --- | --- |
| No case-mix correction | 2.7 (0.0-33.1) | 23.2 (6.8-39.0) | 50.0 (35.8-66.2) | 63.1 (57.7-77.5) |
| Case-mix correction | 1.5 (1.4-29.0) | 20.2 (5.1-32.6) | 44.5 (28.5-57.7) | 59.8 (53.2-70.4) |
| Case-mix correction means adjustment for age, income, tumor grade, multifocality, pT, pN | | | | |

**Supplementary Table 3** Association between various characteristics and the chance of receiving 5 fractions.

|  | 2020–2023 | | 2020–2022 | | | 2023 | | |
| --- | --- | --- | --- | --- | --- | --- | --- | --- |
|  | Unadjusted OR, 95% CI | Adjusted OR, 95% CI^a^ | | Unadjusted OR, 95% CI | Adjusted OR, 95% CI^a^ | | Unadjusted OR, 95% CI | Adjusted OR, 95% CI^a^ |
| **Age** |  |  | |  |  | |  |  |
| 18-49 | **0.24 (0.21-0.27)** | **0.31 (0.27-0.35)** | | **0.35 (0.30-0.41)** | **0.52 (0.44-0.62)** | | **0.10 (0.08-0.13)** | **0.13 (0.10-0.17)** |
| 50-75 | Reference | Reference | | Reference | Reference | | Reference | Reference |
| >75 | **1.43 (1.28-1.61)** | **2.11 (1.81-2.47)** | | **1.31 (1.13-1.52)** | **1.96 (1.62-2.37)** | | **1.68 (1.33-2.12)** | **2.63 (1.99-3.48)** |
| **Income** |  |  | |  |  | |  |  |
| Low | Reference | Reference | | Reference | Reference | | Reference | Reference |
| Middle | **0.89 (0.82-0.97)** | 0.95 (0.85-1.06) | | **0.85 (0.77-0.95)** | 0.97 (0.85-1.11) | | 0.94 (0.80-1.10) | 0.90 (0.74-1.09) |
| High | 0.98 (0.90-1.06) | **0.90 (0.80-1.00)** | | 0.99 (0.89-1.10) | 0.91 (0.80-1.04) | | **0.91 (0.77-1.06)** | 0.85 (0.70-1.04) |
| **Tumour grade** |  |  | |  |  | |  |  |
| 1 | Reference | Reference | | Reference | Reference | | Reference | Reference |
| 2 | **0.70 (0.65-0.76)** | 0.96 (0.86-1.06) | | **0.69 (0.63-0.76)** | 1.01 (0.89-1.14) | | **0.62 (0.53-0.72)** | 0.86 (0.71-1.05) |
| 3 | **0.38 (0.35-0.42)** | **0.70 (0.61-0.80)** | | **0.39 (0.35-0.44)** | **0.87 (0.74-1.02)** | | **0.30 (0.25-0.35)** | **0.45 (0.36-0.58)** |
| **Multifocality** |  |  | |  |  | |  |  |
| No | Reference | Reference | | Reference | Reference | | Reference | Reference |
| Yes | **0.57 (0.50-0.64)** | **0.75 (0.64-0.86)** | | **0.50 (0.42-0.59)** | **0.75 (0.62-0.91)** | | **0.52 (0.43-0.63)** | **0.78 (0.62-0.97)** |
| **(y)pT^b^** |  |  | |  |  | |  |  |
| is | **0.62 (0.53-0.69)** | **0.95 (0.81-1.12)** | | **0.64 (0.56-0.73)** | 0.85 (0.71-1.02) | | **0.68 (0.55-0.84)** | 1.40 (0.98-2.01) |
| 0 | **0.60 (0.53-0.69)** | **1.68 (1.42-1.99)** | | **0.63 (0.53-0.74)** | **1.49 (1.22-1.83)** | | **0.68 (0.53-0.88)** | **2.29 (1.66-3.17)** |
| 1 | Reference | Reference | | Reference | Reference | | Reference | Reference |
| 2 | **0.57 (0.52-0.62)** | **0.83 (0.74-0.94)** | | **0.52 (0.46-0.59)** | 0.86 (0.74-1.01) | | **0.56 (0.48-0.65)** | **0.77 (0.64-0.93)** |
| 3 | **0.40 (0.27-0.59)** | 1.02 (0.63-1.65) | | **0.50 (0.31-0.81)** | 1.33 (0.75-2.36) | | **0.25 (0.12-0.49)** | 0.63 (0.27-1.46) |
| **(y)pN** |  |  | |  |  | |  |  |
| 0 | Reference | Reference | | Reference | Reference | | Reference | Reference |
| 1 | **0.41 (0.35-0.48)** | **0.69 (0.57-0.84)** | | **0.37 (0.30-0.46)** | **0.62 (0.48-0.80)** | | **0.45 (0.34-0.58)** | 0.81 (0.59-1.10) |
| **RT treatment indication** |  |  | |  |  | |  |  |
| WBI | Reference | Reference | | Reference | Reference | | Reference | Reference |
| Thoracic wall | **0.55 (0.41-0.73)** | **0.62 (0.44-0.89)** | | 0.71 (0.50-1.00) | 0.71 (0.46-1.09) | | **0.39 (0.24-0.65)** | **0.45 (0.24-0.86)** |
| PBI | **8.79 (8.02-9.64)** | **8.66 (7.71-9.73)** | | **8.89 (8.00-9.89)** | **8.92**  **(7.81-10.20)** | | **20.26**  **(15.22-26.97)** | **13.17**  **(9.66-17.96)** |
| **Type of RT institution** |  |  | |  |  | |  |  |
| Independent | Reference | Reference | | Reference | Reference | | Reference | Reference |
| General | 0.84 (0.77-0.92) | 0.87 (0.78-0.97) | | 1.06 (0.94-1.19) | 0.91 (0.78-1.05) | | 0.93 (0.80-1.08) | 0.95 (0.78-1.14) |
| Academic | **1.71 (1.59-1.84)** | **2.31 (2.09-2.55)** | | **2.73 (2.47-3.01)** | **3.09 (2.73-3.50)** | | **1.37 (1.20-1.56)** | **1.24 (1.05-1.47)** |
| **Start year of RT** |  |  | |  |  | |  |  |
| 2020 | Reference | Reference | | Reference | Reference | |  |  |
| 2021 | **1.61 (1.43-1.81)** | **1.54 (1.34-1.78)** | | **1.61 (1.43-1.81)** | **1.57 (1.36-1.81)** | |  |  |
| 2022 | **3.45 (3.09-3.86)** | **3.55 (3.10-4.08)** | | **3.45 (3.09-3.86)** | **3.64 (3.17-4.17)** | |  |  |
| 2023 | **6.84 (6.12-7.64)** | **9.70**  **(8.44-11.14)** | |  |  | |  |  |
| CI: confidence interval, OR: odds ratio, PBI: partial breast irradiation, PMRT: post-mastectomy radiotherapy, RT: radiotherapy, WBI: whole breast irradiation.  Analyses were performed for 2020–2023, 2020–2022 and 2023, using the complete case dataset.  a. Adjusted for all variables mentioned in the table  b. (y)pT can be 0 after neo-adjuvant therapy and a pathological complete response, or when the tumour is only found in the lymph nodes | | | | | | | | |

**Supplementary Table 4** Reasons mentioned once for not implementing the 5x5.2Gy schedule, per radiotherapy target volume.

|  | WBI | PBI | Thoracic wall |
| --- | --- | --- | --- |
| Prosthesis | 1 |  | 1 |
| T-DM1^1a^ | 1 |  | 1 |
| Chemotherapy right after radiotherapy | 1 |  | 1 |
| Cross-section | 1 |  | 1 |
| Volume |  | 1 |  |
| Cup size^b^ | 1 |  |  |
| Two operations | 1 |  |  |
| Operation effect | 1 |  |  |
| Protons |  |  | 1 |
| Direct reconstruction |  |  | 1 |
| a. T-DM1 is an adjuvant HER2-targeted therapy.  b. The follow-up question regarding cup size indicated that, depending on age, longer schedules are used for larger cup sizes. | | | |
